# The Risk of Heart Failure and Cardiometabolic Complications in Obesity May Be Masked by an Apparent Healthy Status of Normal Blood Glucose

**DOI:** 10.1155/2013/253657

**Published:** 2013-12-14

**Authors:** Shuchita Tiwari, Manish Mishra, Ashok Jadhav, Courtney Gerger, Paul Lee, Lynn Weber, Joseph Fomusi Ndisang

**Affiliations:** ^1^Department of Physiology, University of Saskatchewan College of Medicine, 107 Wiggins Road, Saskatoon, SK, Canada S7N 5E5; ^2^Department of Veterinary Biomedical Sciences, University of Saskatchewan, 52 Campus Drive, Saskatoon, SK, Canada S7N 5E5

## Abstract

Although many obese individuals are normoglycemic and asymptomatic of cardiometabolic complications, this apparent healthy state may be a misnomer. Since heart failure is a major cause of mortality in obesity, we investigated the effects of heme-oxygenase (HO) on heart failure and cardiometabolic complications in obese normoglycemic Zucker-fatty rats (ZFs). Treatment with the HO-inducer, hemin, reduced markers of heart failure, such as osteopontin and osteoprotegerin, abated left-ventricular (LV) hypertrophy/fibrosis, extracellular matrix/profibrotic proteins including collagen IV, fibronectin, TGF-*β*1, and reduced cardiac lesions. Furthermore, hemin suppressed inflammation by abating macrophage chemoattractant protein-1, macrophage-inflammatory protein-1 alpha, TNF-*α*, IL-6, and IL-1*β* but enhanced adiponectin, atrial-natriuretic peptide (ANP), HO activity, insulin sensitivity, and glucose metabolism. Correspondingly, hemin improved several hemodynamic/echocardiographic parameters including LV-diastolic wall thickness, LV-systolic wall thickness, mean-arterial pressure, arterial-systolic pressure, arterial-diastolic pressure, LV-developed pressure, +dP/dt, and cardiac output. Contrarily, the HO-inhibitor, stannous mesoporphyrin nullified the hemin effect, exacerbating inflammatory/oxidative insults and aggravated insulin resistance (HOMA-index). We conclude that perturbations in insulin signaling and cardiac function may be forerunners to overt hyperglycemia and heart failure in obesity. Importantly, hemin improves cardiac function by suppressing markers of heart failure, LV hypertrophy, cardiac lesions, extracellular matrix/profibrotic proteins, and inflammatory/oxidative mediators, while concomitantly enhancing the HO-adiponectin-ANP axis.

## 1. Introduction

The recent escalation of obesity in every segment of the population including children, adolescences, and adults poses a great health challenge of considerable socioeconomic burden [1, 2]. The impact on healthcare systems may become unsustainable given the numerous chronic diseases such as type-2 diabetes, dyslipidemia, hypertension, and other related cardiometabolic complications associated with obesity [1–3]. Cardiac complications including heart failure are among the major causes of mortality in obese individuals. Obesity causes lipotoxicity and adipose tissue dysfunction with excessive production of adipokines like tumor necrosis factor-*α* (TNF-*α*) and interleukin 6 (IL-6) IL-1*β*, all of which are implicated in heart failure and related cardiometabolic complications [4, 5]. However, obesity may not always translate into increased risk for these comorbidities [6]. Some obese individuals dubbed “metabolically healthy” are protected against obesity-related metabolic diseases. These “metabolically healthy” obese individuals are insulin sensitive with normal lipid metabolism and cardiac function similar to healthy lean individuals, which is in stark contrast to “metabolically unhealthy” obese individuals with high risk of developing cardiometabolic complications [6]. However, the apparent state of good health in “metabolically healthy” obese subphenotype may be a misnomer because the development of several characteristics of metabolic syndrome is now being observed in many adults who previously manifested the healthy obese phenotype [7], suggesting that individuals with a healthy obese phenotype may not remain healthy for their entire lives. Several parameters including environmental and behavioral factors may modify obesity subphenotypes, and the transition from healthy to unhealthy. Whether healthy obese individuals can maintain insulin sensitivity during the entire life or whether healthy obesity simply represents delayed onset of obesity related cardiometabolic complications has to be clarified.

In obesity, excessive oxidative stress, intense inflammatory activity, insulin resistance, deregulated lipid metabolism, altered glucose metabolism, and impaired mitochondrial biogenesis are among the pathophysiological driving forces that precede the early stages of cardiac dysfunction. Many cardiac complications have the common denominator of elevated inflammation due to the infiltration of macrophage M1 phenotype [8]. Generally, macrophages exhibit two different forms dubbed “classical” or M1 phenotype and “alternative” or M2 phenotype [8], and each phenotype expresses distinct patterns of surface receptors when responding to different stimuli. The M1 phenotype stimulates inflammation while the M2 phenotype blunts inflammation [8]. During macrophage infiltration, the M1 phenotype is stimulated by different chemokines including macrophage inflammatory protein-1 alpha ((MIP-1*α*), chemokine (C-C motif) ligand-3 (CCL3)) and macrophage chemoattractant protein-1 ((MCP-1) [9], chemokine (C-C motif ligand-2 (CCL2)) [9]. The activation of the macrophage M1 phenotype is generally associated with elevated levels of proinflammatory cytokines including TNF-*α*, IL-6, and IL-1*β* [10–12]. Moreover, the levels of macrophage M1 phenotype, MCP-1, TNF-*α*, IL-6, and IL-1*β* are elevated in obesity and insulin resistance [9, 10, 13], and these factors play a major pathophysiological role in heart failure [4]. In obesity, markers of heart failure such as osteopontin [14] and osteoprotegerin [15] are elevated [16, 17]. Similarly, the levels of extracellular matrix/profibrotic proteins like transforming growth factor beta (TGF-*β*), collagen, and fibronectin are elevated in obesity [18]. Therefore, in obesity elevated chemokines, cytokines and increased macrophage-M1 infiltration would act in concert with elevated extracellular matrix/profibrotic and heart-failure proteins to exacerbate cardiac tissue destruction and compromise heart function. Thus, novel strategies capable of selectively suppressing macrophage M1 phenotype, proinflammatory cytokines/chemokines, and extracellular matrix/profibrotic proteins are needed.

In many pathophysiological conditions, various stress-response immune-regulatory proteins, including heme oxygenase (HO-1), are activated as an innate defense mechanism [19–22]. However, the pathophysiological activation of HO-1 may only result in a transient or marginal increase of HO activity that falls below the threshold necessary to activate the downstream signaling components through which the HO system elicits its cytoprotective effects, so a robust and surmountable increase of HO activity with HO inducers like hemin may be needed for cardioprotection [23–27]. Generally, HO is composed of two main isoforms (HO-1 or inducible) and (HO-2 or constitutive), which are largely responsible for the antioxidant and anti-inflammatory effects of HO [28]. We recently reported the cardioprotective effects of the HO system in Zucker diabetic fatty rats (ZDFs) [13], a model characterized by obesity, insulin resistance, and overt hyperglycemia. However, because ZDFs are hyperglycemic, their pathophysiological profile is not reflective of individuals dubbed “metabolically healthy,” a subtype of obesity characterized by normoglycemia [7]. Given that the incidence of cardiometabolic complications is increasing in many adults who previously manifested the metabolically healthy obese phenotype [7], novel studies with animal models that closely mimic the pathophysiological profile of metabolically healthy obese subtype are needed. Therefore, this study will investigate the effects of the HO system on cardiometabolic complications in Zucker fatty rats (ZFs), an obese model with normoglycemia and cardiometabolic complications [29] that closely mimic the pathophysiological profile of metabolically healthy obese individuals with normoglycemia and an apparent state of good health. Although the HO system is cytoprotective, its effects on cardiomyopathy in ZFs remain to be elucidated.

Since dysfunctional insulin signaling, obesity, elevated inflammation, and cardiac hypertrophy are forerunners to heart failure, this study will also investigate the multifaceted mechanisms by which the HO system preserves cardiac function in ZFs. Whether an upregulated HO system by hemin is capable of modulating macrophage polarization towards the M2 phenotype that blunts inflammation, while suppressing the proinflammatory M1 phenotype, will be assessed. As macrophage infiltration is stimulated by chemokines like MIP-1*α* and MCP-1 [9], and the effects of the HO system on these chemokines in ZFs have not been reported, this study will also determine left-ventricular MIP-1*α* and MCP-1 and correlate changes of these chemokines to the expression of the proinflammatory macrophage-M1 phenotype in the left ventricle of ZFs. Similarly, the effect of hemin therapy on important markers of heart failure such as osteopontin [13] and osteoprotegerin [14] will be investigated. Importantly, no study has reported the levels of expression of osteopontin and osteoprotegerin in myocardial tissue of ZFs. Therefore this study will unveil the multifaceted mechanisms by which hemin therapy improves cardiac function and insulin signaling in obesity.

## 2. Materials and Methods

### 2.1. Animals, Treatment Groups, and Biochemical Parameters

Our experimental protocol was in conformity with the Guide for Care and Use of Laboratory Animals stipulated by the Canadian Council on Animal Care and the National Institutes of Health (NIH Publication no. 85-23, revised 1996) and was approved by University of Saskatchewan Animal Ethics Committee. Male ZFs (12 weeks old) and sex/age-matched Zucker lean (ZL) rats were purchased from Charles River Laboratories (Willington, MA, USA). The animals were housed at 21°C with 12-hour light/dark cycles, fed with standard laboratory chow, and had access to drinking water *ad libitum*.

The HO-inducer, hemin (30 mg/kg i.p., Sigma, St Louis, MO, USA), and HO-blocker stannous-mesoporphyrin ((SnMP) 2 mg/100 g body weight i.p.) were purchased from Porphyrin Products (Logan, UT, USA), and prepared as we previously reported and administered biweekly for 8 weeks [13, 30, 31]. At 16 weeks of age, the animals were randomly assigned to the following experimental groups (*n* = 6 per group): (A) controls (ZF and ZL), (B) hemin-treated ZF and ZL, (C) ZF+hemin+SnMP, and (D) ZF+vehicle dissolving hemin and SnMP.

During the treatment period body weight and glucose were monitored on a weekly routine. Body weight was measured using a digital balance (Model *Mettler PE1600*, *Mettler* Instruments Corporation, Greifensee, Zurich, Switzerland). At the end of the 8-week treatment period, the animals were 24 weeks of age. A day prior to killing, the animals were fasted in metabolic cages for 24 hr urine collection and weighed. Systolic blood pressure was determined by noninvasive tail-cuff method (Model 29-SSP, Harvard Apparatus, Montreal, Canada), while a Millar Mikro-Tip ultra-miniature tip sensor pressure transducer catheter (Model SPR-407, Harvard Apparatus, Montreal,Canada) for invasive hemodynamic parameters. In addition, a Vevo 660 high frequency ultrasound machine (VisualSonics, Markham, ON, Canada) *equipped with B-mode imaging* was used for echocardiographic measurements as we previously reported [13]. After anaesthetizing the animals with pentobarbital sodium (50 mg/kg i.p.), blood was collected by cardiac puncture, and the heart was cleaned and weighed with an analytical balance (Precisa Instruments Ltd., Dietikon, Switzerland) as we previously reported [32]. The atria were removed from the heart and the right ventricle free wall separated from the left ventricle including the septum as we previously reported [32].

Left-ventricular HO activity was evaluated spectrophotometrically as we previously reported [30, 32]. ELISA kits were used for HO-1 (Stressgen-Assay Design, Ann Arbor, MI, USA), adiponectin (Phenix Pharmaceuticals, Inc., Burlingame, CA, USA), TNF-*α*, IL-6, and IL-1*β* (Immuno-Biological Laboratories Co Ltd., Gunma, Japan), MIP-1*α*, and MCP-1 (OmniKine, Assay Biotechnology Company Inc., Sunnyvale, CA, USA) [33, 34], while EIA kits for 8-isoprostane, ANP, ET-1, cGMP, and kits for cholesterol and triglyceride were purchased from Cayman (Cayman Chemical, Ann Arbor, MI, USA) following the manufacturers' instructions as we reported [13, 30, 32]. Intraperitoneal glucose tolerance test (IPGTT) and homeostasis model assessment of insulin resistance (HOMA-IR) were done as we previously reported [30].

### 2.2. Histological, Morphological, and Immunohistochemical Analyses of Left Ventricle

Histological and morphometric analyses were done as we previously described [35]. Sections of 5 *μ*m were cut and stained with hematoxylin and eosin for histological analysis. Masson's Trichrome staining detected left-ventricular collagen deposition. Morphometrical evaluation of left-ventricular longitudinal myocyte thickness was done by randomly measuring 30 cardiac muscle fibers from each experimental group by a blinded researcher using a microscope (Aperio Scan Scope Model CS, Aperio Technologies Inc., Vista, CA, USA) and analyzed using Aperio Image Scope V11.2.0.780 software (Aperio, e-Pathology Solution, Vista, CA, USA). Morphologic assessment of collagen deposition in left-ventricular sections was accessed using Aperio ImageScope (Aperio Technologies Inc., Vista, CA, USA). Each left-ventricular section was magnified at 200x, and 20 random snaps were taken per slide (20 × 6 = 120 images per group) subsequently scored semiquantitatively by a blinded researcher as we previously reported [13, 35].

Immunohistochemistry was done as we previously reported [36]. Sections of 5 *μ*m of left-ventricular tissue were treated with bovine serum albumin in phosphate-buffered saline to block nonspecific staining and incubated overnight with ED1 (1 : 500 dilution, sc-59103, Santa Cruz Biotechnology, CA, USA). The sections were later treated with with goat anti-mouse IgG for 30 min (1 : 200 dilution; Jackson Immuno-Research Laboratories, Inc., ME, USA). Immunohistochemical staining was done using the standard avidin-biotin complex method with the chromagen 3,3′-diaminobenzidine (DAB) at the final detection step. Sections of heart tissue were scanned using virtual microscope (Aperio Scan Scope Model CS, Aperio Technology Inc., Vista, CA, USA). Quantitative assessment of ED1 was done by a blinded researcher who randomly examined 20–22 fields of each left-ventricular section magnified at 200x. Macrophages which were positively stained with ED1 (brown from immune-stained sections) were quantified by manually counting the ED1-stained cells around the blood vessels and interstitial spaces of myocardium.

### 2.3. Western Immunoblotting

Left-ventricular tissue was homogenized as previously reported [13, 30–32, 35]. Primary antibodies ((Santa Cruz Biotechnology, Santa Cruz, CA, USA), ED-1 (CD68) (sc-59103), ED-2 (CD163) (sc-58956), CD-14 (sc-9150), CD-206 (sc-48758), CD-36 (sc-9154), osteopontin (sc-21742), osteoprotegerin (sc-11383), PI3K (sc67306), IRS-1 (sc-559), collagen IV (sc-11360), fibronectin (sc-18825), TGF-*β*1/2/3 (sc7892)) and GLUT4 (ab 654, Abcam Inc., Cambridge, MA, USA) were used. Densitometric analysis was done with UN-SCAN-IT software (Silk Scientific Inc., Orem, UT, USA). G6PDH antibody (A9521, Sigma St. Louis, MO, USA) was used as a control to ascertain equivalent loading.

### 2.4. Statistical Analysis

All data are expressed as means ± SEM from at least four independent experiments unless otherwise stated. Statistical analyses were done using two-way ANOVA, using Statistical Analysis System (SAS), software Version 9.3 (SAS Institute Inc., Cary, NC, USA) and Student's *t*-test. Group differences at the level of *P* < 0.05 were considered statistically significant.

## 3. Results

### 3.1. Hemin Therapy Upregulates the HO System to Improve Cardiac Function

**Figure 1:**
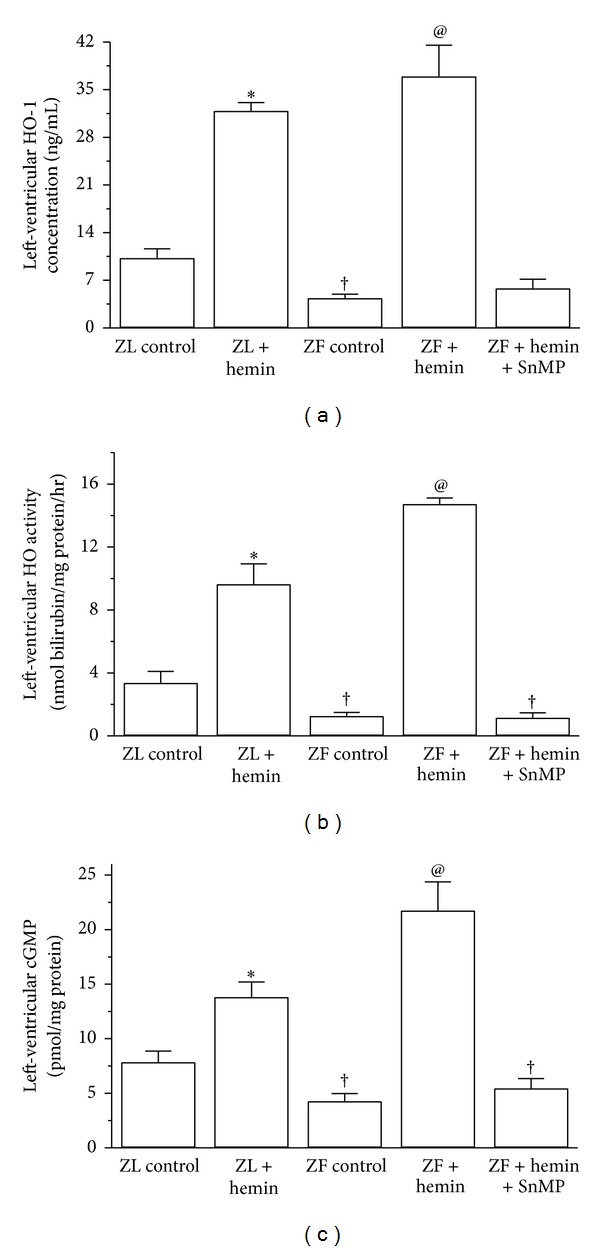
Effects of the HO inducer, hemin, and the HO inhibitor, SnMP on HO-1, HO activity and cGMP in the left ventricle of ZLs and ZFs. (a) Hemin enhanced HO-1, whereas SnMP nullified the effects of hemin. (b) Hemin increased HO activity, while SnMP abolished the hemin effect. (c) Hemin enhanced cGMP, which, however, was abolished by SnMP. Bars represent means ± SEM; *n* = 6 rats per group (*P < 0.01 versus ZL control; ^†^ *P* < 0.05 versus Z control; ^@^ *P* < 0.01 versus ZF+hemin+SnMP or ZF control).

To investigate the mechanisms underlying the improvement of cardiac function in obese insulin-resistant ZFs, we measured the concentration of HO-1 and HO activity. In ZF-control rats, the basal level of HO-1 concentration and HO activity was significantly lower than that of ZL control (Figures 1(a) and 1(b)). However, hemin administration increased HO-1 and HO activity in ZF by 8.4- and 11.3-fold, respectively. The enhanced HO activity would increase endogenous carbon monoxide that would in turn stimulate cGMP [30, 32]. Both cGMP and carbon monoxide are known to enhance insulin signaling and glucose metabolism [37]. Accordingly, we detected a 3.4-fold increase of cGMP in hemin-treated animals (Figure 1(c)). In contrast, the coadministration of the HO blocker, SnMP and the HO inducer, and hemin abolished the hemin-induced increase of HO-1 and HO activity, with corresponding reduction of cGMP levels (Figure 1(c)). Hemin therapy also enhanced HO-1, HO activity, and cGMP levels in ZL-control rats (Figures 1(a), 1(b), and 1(c)). In hemin-treated ZLs, HO-1, HO activity and cGMP were enhanced by 3.1-, 2.8-, and 2.4-fold, respectively, as compared to 8.4-, 11.3-, and 3.4-fold, respectively, in hemin-treated ZFs, suggesting greater selectivity of hemin to the HO system in unhealthy ZFs characterized obesity, insulin resistance, and cardiomyopathy [29].

Since cardiac hypertrophy is amongst the forerunners to heart failure, we investigated the effects of hemin on cardiac hypertrophy. Our results indicate that hemin therapy significantly reduced cardiac hypertrophy in ZF, whereas the coadministration of hemin and SnMP nullified the effect (Table 1). Echocardiography was used to further assess left-ventricular hypertrophy. Our hemodynamic data obtained during catheterization of the left side of hearts from ZFs showed association between elevated myocardial hypertrophic response and obesity. Asignificant 2-fold increase in left-ventricular free wall thickness, an important index of cardiac hypertrophy [38], was observed during diastole and systole, and interestingly these were abated by hemin by 33.3% and 15.6%, respectively (Table 2). Other hemodynamic deficiencies in ZFs including abnormalities in left-ventricular end-diastolic volume, left-ventricular end-systolic volume, stroke volume, and cardiac output which were reduced by 17.5%, 16%, 8.3%, and 7.7%, respectively (Table 2), were increased by hemin therapy by 15.2%, 27.3%, 13.6% and 12.4%, respectively. Hemin therapy also improved cardiac hemodynamics by lowering arterial-systolic pressure, arterial-diastolic pressure, mean-arterial pressure, and total-peripheral resistance by 12.4%, 11.4% and 12.2%, 17.6%, respectively, with corresponding reduction of +dp/dt (the maximal rate of increase in left-ventricular pressure), left-ventricular developed pressure, and heart rate.

Treatment with hemin and SnMP caused loss of body weight in ZL controls and ZFs, which, however, did not exceed 9% (Table 1). The loss of weight may not be due to toxicity as we recently showed that several indices of toxicity including plasma gamma-glutamyltransferase, aspartate aminotransferase, and alanine aminotransferase were within normal range [30]. Although ZFs had normoglycemia, hemin and SnMP affected blood glucose. In hemin-treated animals, there was a slight but significant reduction of glycemia, whereas in SnMP-treated animals a slight increase was observed (Table 1). Similarly, hemin therapy slightly reduced glycemia in ZL controls. The vehicle dissolving hemin and SnMP had no effect on the measured parameters.

### 3.2. Hemin Therapy Abates MCP-1, MIP-1*α*, TNF-*α*, Endothelin-1, and 8-Isoprostane but Enhanced ANP in ZFs

**Figure 2:**
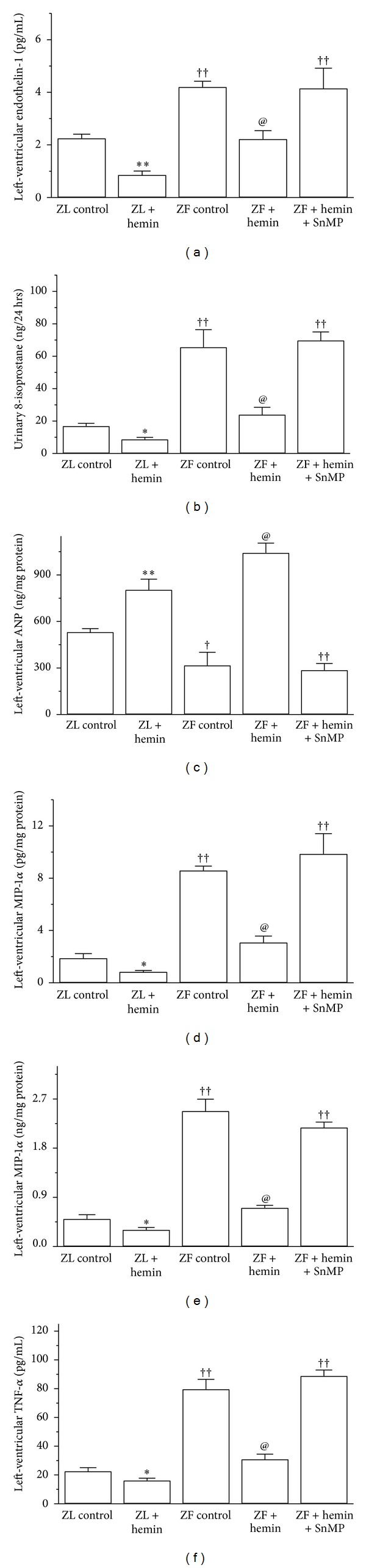
Effects of the HO inducer, hemin and the HO inhibitor, SnMP on endothelin-1, ANP, MCP-1, MIP1-*α*, and TNF-*α* in left-ventricular tissue from ZLs and ZFs. Hemin therapy: (a) reduced endothelin-1, (b) attenuated 8-isoprostane, (c) increased ANP, (d) suppressed MCP-1, (d) abated MIP-1*α*, and (f) reduced TNF-*α*, whereas SnMP abolished the hemin effects. Bars represent means ± SEM; *n* = 6 rats per group (*P < 0.05, **P < 0.01 versus ZL control; ^†^ *P* < 0.05, ^††^ *P* < 0.01 versus ZL control; ^@^ *P* < 0.01 versus ZF+hemin+SnMP or ZF control).

Since 8-isoprostane stimulates ET-1 [39], and both ET-1 and 8-isoprostane are involved in the oxidative destruction of tissue, we measured ET-1 and 8-isoprostane. ET-1 in untreated ZFs was markedly elevated as compared to ZL controls (Figure 2(a)) but was significantly abated by hemin. In contrast, the coadministration of hemin and the HO blocker, SnMP, annulled the effect of hemin (Figure 2(a)). Because elevated oxidative stress is linked to impaired insulin-signaling and cardiac dysfunction, we measured urinary 8-isoprostane, an important marker of oxidative stress [40]. In ZFs, the basal levels of 8-isoprostane were significantly elevated (Figure 2(b)) but were reduced by hemin, whereas cotreatment of hemin with SnMP nullified the effects. Given that ET-1 and ANP are known to interact reciprocally [41], we investigated whether the hemin-dependent suppression of ET-1 (Figure 2(a)) would be associated with a parallel potentiation of ANP. In ZFs, the basal ANP levels were markedly depressed by 1.7-fold (Figure 2(c)) but interestingly were robustly enhanced by hemin by 3.3-fold. In contrast, the coadministration of hemin with SnMP abolished the effects of hemin.

We also investigated the effects of hemin on MIP-1*α* and MCP-1 since these chemokines trigger macrophage infiltration [9]. In ZFs, the basal MCP-1 levels were significantly increased by 4.6-fold (Figure 2(d)) but were attenuated by hemin by 2.8-fold, whereas the coadministration with SnMP nullified the effects of hemin (Figure 2(d)). Although hemin therapy greatly attenuated MCP-1 by 64% in ZF, however comparable levels as observed in the ZL controls were not reinstated. Hemin therapy was also effective in suppressing MIP-1*α* (Figure 2(e)). In ZFs, the basal MIP-1*α* levels were significantly elevated by 4.9-fold but were reduced by hemin by 3.5-fold, whereas the cotreatment of hemin with SnMP nullified the effects (Figure 2(e)). Since TNF-*α* is implicated in macrophage infiltration [9], we also assessed the effects of hemin on TNF-*α*. In ZFs, the basal levels of TNF-*α* were elevated by 3.5-fold but were significantly attenuated by hemin by 2.6-fold (Figure 2(f)), whereas cotreatment with SnMP abolished the effect of hemin.

Hemin therapy also affected ET-1, 8-isoprostane, ANP, MCP-1, and MIP-1*α* in ZL controls although the magnitude of effect was smaller than that in ZFs (Figure 2).

### 3.3. Hemin Selectively Abated the Proinflammatory Macrophage M1 Phenotype but Enhanced the Anti-Inflammatory M2 Phenotype in the Left Ventricle of ZFs

After having observed the hemin-dependent reduction of cytokines/chemokines implicated in macrophage infiltration such as MIP-1*α*, MCP-1, and TNF-*α*, we investigated whether the suppression of these chemokines/cytokines in the left ventricle of ZFs would be accompanied by the selective attenuation of the proinflammatory macrophage M1 phenotype using a specific marker such as ED1 [42] to quantify the expression of the proinflammatory M1 phenotype in left-ventricular tissue and other markers for the assessment of anti-inflammatory M2 phenotype including ED2 [42], CD14 [43, 44], CD206 [8], and CD36 [45, 46].

**Figure 3:**
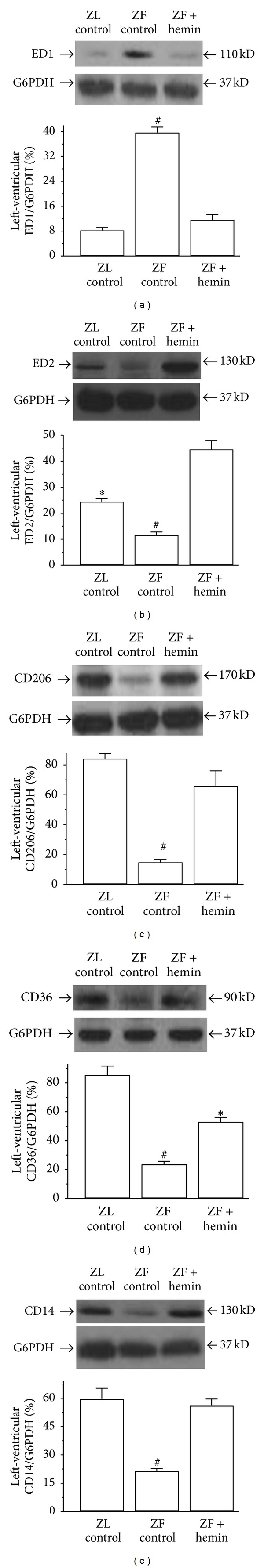
Effects of hemin on ED1, ED2, CD206, CD36, and CD14 in left-ventricular tissue from ZLs and ZFs. Hemin therapy (a) abated ED1, but (b) enhanced ED2, (c) increased CD206, (d) enhanced CD36, and (e) increased CD14 in ZFs. Bars represent means ± SEM; *n* = 4 rats per group (*P < 0.01 versus all groups; ^#^ *P* < 0.01 versus all groups).

Our Western immunoblotting and relative densitometry revealed that the basal expression of the proinflammatory macrophage M1 phenotype marker, ED1, in ZFs was markedly elevated by 4.8-fold as compared to ZL controls (Figure 3(a)) but was significantly reduced by hemin by 3.5-fold although control levels were not attained. On the other hand, the basal expression of several markers of the anti-inflammatory macrophage M2 phenotype including ED2, CD206, CD36, and CD14 was significantly depressed in ZFs by 2.1-, 5.7-, 3.6-, and 2.9-fold, respectively, (Figures 3(b), 3(c), 3(d), and 3(e)). Interestingly, hemin therapy greatly enhanced the depressed ED2, CD206, CD36, and CD14 in ZFs by 3.8-, 4.1-, 2.3-, and 2.6-fold, respectively, suggesting that a novel mechanism by which hemin therapy blunts inflammation is by selectively modulating the polarization of macrophage toward the M2 phenotype that dampens inflammation.

### 3.4. Hemin Therapy Suppressed Macrophage Infiltration in the Left Ventricle of ZFs

**Figure 4:**
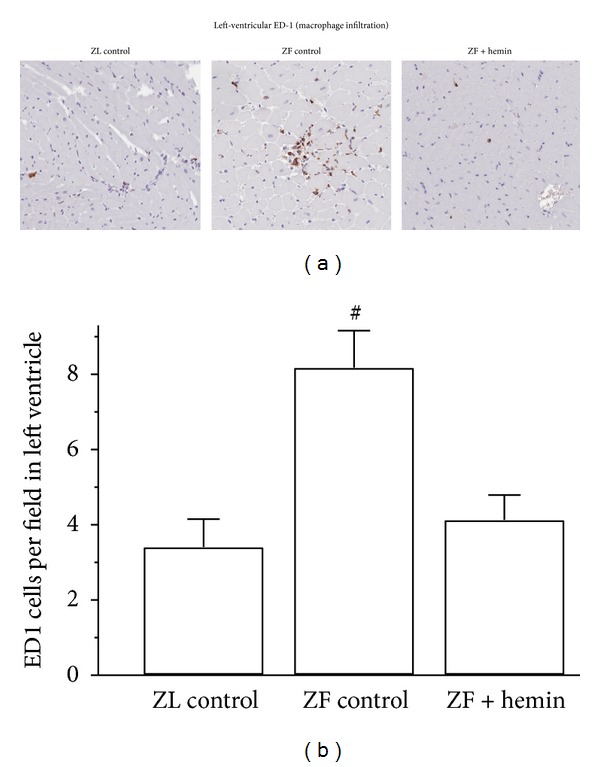
(a) Representative photomicrographs of cross-sections of the left-ventricle showing macrophage infiltration (ED1-positive cells stained dark brown) (magnification ×200). (b) Quantitative analyses of macrophage infiltration per field indicating that in ZFs the number of ED1-positive dark-brown cells (macrophage infiltration) was markedly elevated as compared to ZL control but interestingly was significantly attenuated by hemin therapy. Bars represent means ± SEM; *n* = 6 rats per group (^#^ *P* < 0.01 versus all groups).

Following the observation from our Western blot experiment that hemin therapy reduced left-ventricular ED-1, a marker of macrophage infiltration, we use the ED-1 antibody to further confirm macrophage infiltration in the left ventricle by immunohistochemistry (Figure 4(a)). Our results reveal that left-ventricular sections from ZL-control rats were almost devoid of the dark brown ED1 positive staining. However, in untreated ZF-control rats, a greater number of ED1-positively stained dark brown cells were observed, indicating increased macrophage infiltration. Interestingly, in hemin-treated ZFs, there was a marked reduction in the number of dark brown-stained macrophages, suggesting reduction of macrophage infiltration. Correspondingly, hemin therapy significantly reduced the quantitative ED1 score (Figure 4(b)).

### 3.5. Hemin Therapy Enhanced Insulin Signaling but Suppressed Extracellular Matrix and Profibrotic Proteins Implicated in Cardiac Injury

**Figure 5:**
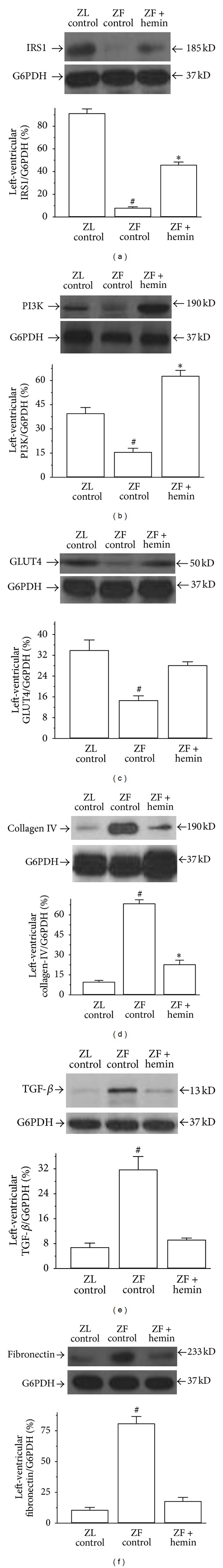
Effects of hemin on the expression of important proteins of the insulin signal transduction pathway such as IRS-1, PI3K, GLUT4, and the expression of profibrotic/extracellular matrix proteins including collagen IV, TGF-*β*, and fibronectin in left-ventricular tissue from ZLs and ZFs. Representative Western immunoblotting and relative densitometry of the expressed proteins normalized by G6PHD indicates that hemin therapy significantly (a) enhanced IRS-1, (b) increased PI3K, (c) upregulated GLUT4, but (d) abated collagen IV, (e) reduced TGF-*β*, and (f) suppressed fibronectin in ZFs. Bars represent means ± SEM; *n* = 4 rats per group (*P < 0.01 versus all groups; ^#^ *P* < 0.01 versus all groups).

Since visceral adiposity and elevated inflammation impair insulin signaling [47], we investigated the effects of hemin therapy on the expression of important components of the insulin signal transduction pathway including IRS-1, PI3K, and GLUT4. In in ZFs, the basal expression of IRS-1, PI3K, and GLUT4 was significantly reduced by 11.2-, 2.5-, and 2.3-fold as compared to the ZL control (Figures 5(a), 5(b), and 5(c)) but was enhanced by hemin by 5.7-, 4.01-, and 1.9-fold, respectively.

To further confirm the antihypertrophic effect of hemin therapy, we measured collagen IV, an important protein implicated in cardiac hypertrophy and fibrosis [35]. In ZFs, the basal expression of left-ventricular collagen IV was significantly elevated by 6.9-fold but was abated by hemin by 2.8-fold (Figure 5(d)). Given that excessive deposition of extracellular matrix/profibrotic proteins and inflammation due to macrophage infiltration are cardinal pathophysiological events implicated in cardiac insult [47, 48], while atrial natriuretic peptide (ANP) and adiponectin are known to suppress fibrosis caused by the deposition of extracellular matrix [49, 50], we investigated whether the concomitant potentiation of ANP, adiponectin, and the HO system by hemin would abate TGF-*β*. In ZFs, the basal expression of TGF-*β* was significantly elevated by 4.6-fold but was markedly attenuated by hemin by 3.4-fold (Figure 5(e)). Since TGF-*β* mobilizes the extracellular matrix by stimulating fibronectin and collagen to cause fibrosis and cardiac injury [49, 51], we also measured the expression of fibronectin. In ZF rats, the basal expression of fibronectin was increased by 7.5-fold but was markedly attenuated by hemin therapy by 4.5-fold (Figure 5(f)).

### 3.6. Hemin Improved Glucose Tolerance, Enhanced the Insulin-Sensitizing Protein, Adiponectin, but Abated Insulin Resistance

**Figure 6:**
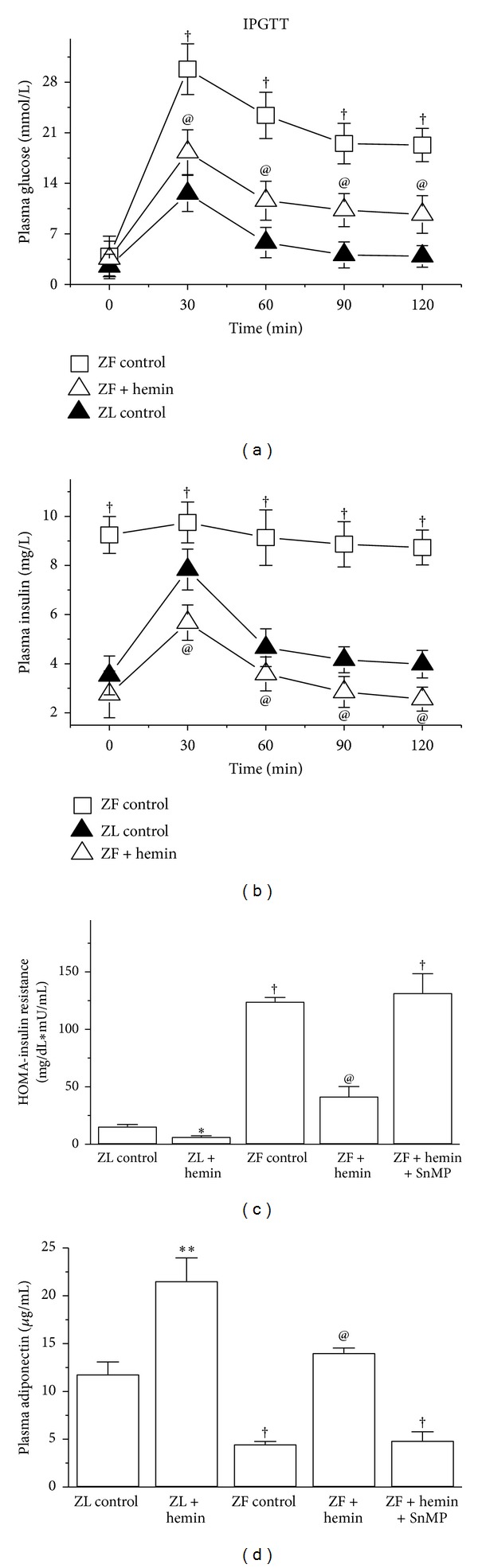
Effects of hemin on glucose tolerance, insulin resistance (HOMA-IR index), and adiponectin. Hemin therapy (a) improved glucose tolerance (IPGTT), (b) increased glucose-stimulated insulin release, (c) reduced insulin resistance, and (d) increased adiponectin. Bars represent means ± SEM; *n* = 6 rats per group (*P < 0.05, **P < 0.01 versus ZL control; ^†^ *P* < 0.01 versus ZL control; ^@^ *P* < 0.01 versus ZF+hemin+SnMP or ZF control).

After having observed the hemin-induced potentiation of insulin signalling, to further confirm the role of hemin therapy on glucose metabolism, we assessed the effects of hemin on glucose tolerance, insulin resistance, and the insulin-sensitizing protein, adiponectin in ZFs, an obese model with elevated inflammation. Since inflammation due to macrophage infiltration is implicated in insulin resistance and cardiomyopathy [47, 48], and ZFs are characterized by insulin resistance [29], we investigated whether the hemin-dependent suppression of macrophage infiltration would be accompanied by improved glucose metabolism. In untreated ZFs, IPGTT analysis showed marked increase in glycemia as compared to ZL controls and hemin-treated ZFs at all time points tested (Figure 6(a)), suggesting improved glucose tolerance in hemin-treated ZFs. Although ZFs were hyperinsulinemic with elevated basal glycemia, when challenged with a bolus injection of glucose, only to a meagre glucose-stimulated insulin release was observed (Figure 6(b)), suggesting glucose intolerance. On the other hand, glucose challenge to ZL controls and hemin-treated ZFs greatly stimulated insulin release (Figure 6(b)), suggesting improved glucose tolerance. Hemin also reduced the elevated insulin resistance HOMA-IR in ZFs (Figure 6(c)), whereas coadministration with SnMP reversed the effects of hemin (Figure 6(c)).

We also investigated the effects of hemin therapy on adiponectin, an anti-inflammatory, insulin sensitizing and cardioprotective protein [52, 53]. Interestingly, hemin therapy significantly enhanced the depressed basal adiponectin levels in ZFs, whereas treatment with SnMP abolished and further reduced the depressed levels of adiponectin (Figure 6(d)). Hemin therapy also reduced HOMA-IR index in ZL controls and enhanced adiponectin although the effect was less intense as compared to ZFs.

### 3.7. Hemin Therapy Suppressed Left-Ventricular Fibrosis, Cardiomyocyte Hypertrophy, and Longitudinal Cardiac Myofibril Thickness in ZFs

**Figure 7:**
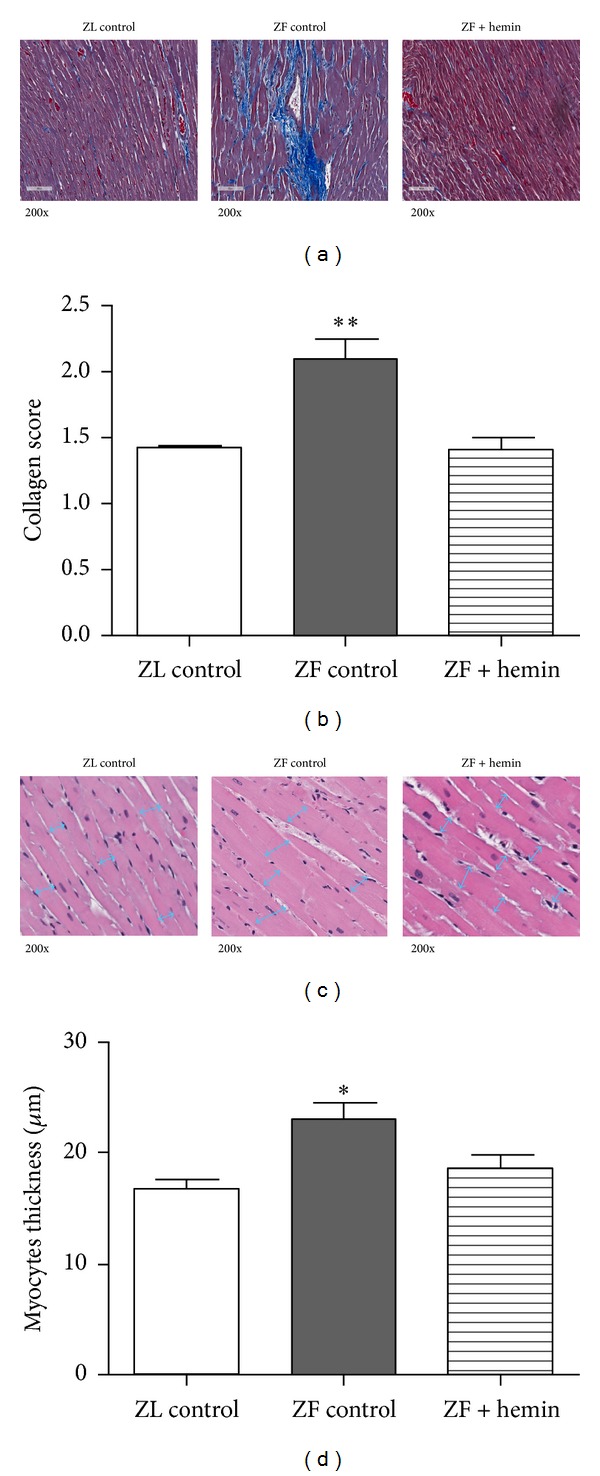
Effect of hemin on histological lesions in the left ventricle of ZLs and ZFs. (a) Representative Mason's trichrome-stained images revealing severe cardiac muscle scaring and collagen deposition in ZFs. (b) Semiquantitative evaluation showed that hemin therapy reduced collagen deposition in ZFs. (c) Representative hematoxylin and eosin-stained images revealing severe longitudinal muscle-fiber thickness in ZFs. (d) Quantitative evaluation showed that hemin reduced longitudinal muscle-fiber thickness. ± SEM; *n* = 6 rats per group (*P < 0.05, **P < 0.01 versus all groups).

Histological and morphometric analyses using Masson's trichrome and hematoxylin and eosin staining were done to further confirm the cardioprotection by hemin. Cardiomyocytes appeared as dark reddish while extracellular matrix, such as collagen, stained blue (Figure 7(a)). Left-ventricular sections from ZL controls appeared morphologically normal, with scanty interstitial collagen deposition. In contrast left-ventricular images from ZFs showed moderate-to-severe fibrosis, with scarring of cardiomyocytes, and interstitial and perivascular collagen depositions (Figure 7(a)). Interestingly, hemin therapy attenuated the severity of scarring and intestinal and perivascular collagen deposition, evidenced by reduced extracellular and perivascular blue staining (Figure 7(a)). Correspondingly, semiquantitative analysis showed that hemin therapy significantly abated the elevated collagen deposition and perivascular fibrosis in ZFs, reinstating comparable levels to ZL control (Figure 7(b)).

Hemin therapy was also effective against cardiomyocyte hypertrophy (Figure 7(c)). In ZFs, cardiomyocytes were enlarged with increscent nuclei and the inner myofibril spaces were decreased, as compared to normal cardiomyocytes in ZL controls (Figure 7(c)). In ZFs, the longitudinal cardiac myofibril thickness was 37% higher than that of ZL controls (Figure 7(d)) but was reduced by 27% in hemin-treated ZFs. Although ZL-control values were not reinstated, hemin increased intermyofibril spaces in ZFs close to the levels observed in ZL controls (Figure 7).

### 3.8. Hemin Therapy Suppressed the Elevated Expression of Markers of Heart Failure in the Left Ventricle of ZFs

**Figure 8:**
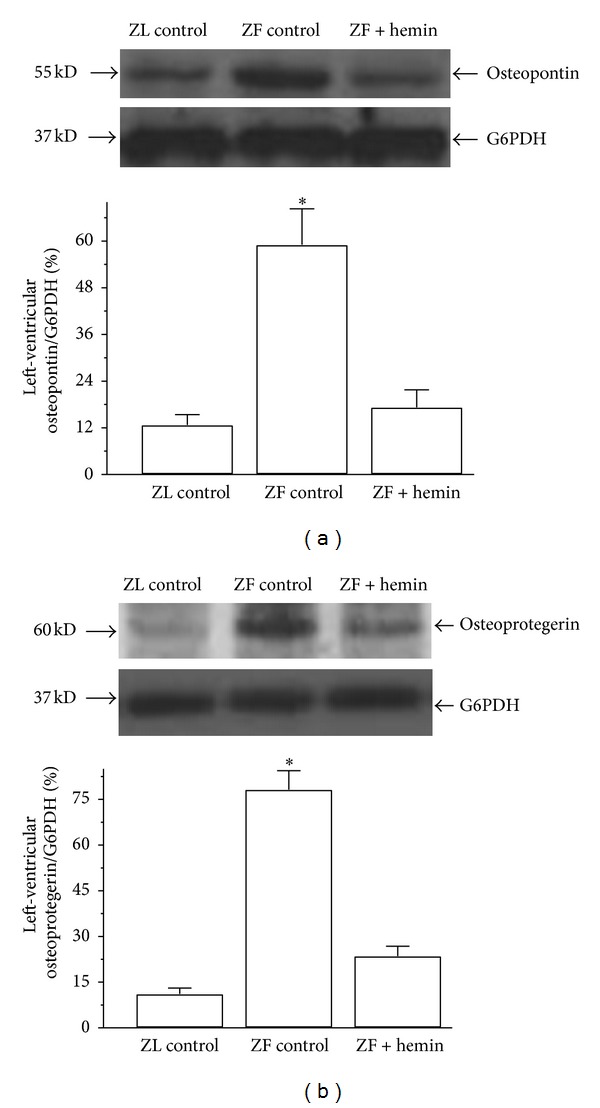
Effect of hemin on markers of heart failure such as osteopontin and osteoprotogerin in the left ventricle of ZLs and ZFs. Representative Western immunoblotting and relative densitometry of the expressed proteins normalized by G6PHD indicates that hemin therapy significantly (a) abated osteopontin and (b) reduced osteoprotogerin in ZFs. Bars represent means ± SEM; *n* = 4 rats per group (*P < 0.01 versus all groups).

To further confirm the cardioprotective effects of an upregulated HO system, we investigated the effects of hemin therapy on important markers of heart failure such as osteopontin [13] and osteoprotegerin [14]. Since left-ventricular hypertrophy is associated with heart failure [54], we determined whether the hemin-dependent suppression of left-ventricular hypertrophy in ZFs would be accompanied by the reduction of markers of heart failure. Our results indicate that, in ZFs, the basal expression levels of osteopontin and osteoprotegerin were significantly elevated by 4.6- and 7.1-fold, respectively, as compared to ZL controls (Figures 8(a) and 8(b)). Interestingly, treatment with hemin attenuated the expressions of osteopontin and osteoprotegerin by 3.5- and 3.3-fold, respectively, (Figures 8(a) and 8(b)).

## 4. Discussion

The present study indicates that the multifaceted mechanisms by which hemin therapy improves cardiomyopathy in obesity include (i) the suppression of visceral adiposity, (ii) the reduction of macrophage M1 phenotype, (iii) the attenuation of markers of heart failure, (iv) the reduction of extracellular matrix/profibrotic proteins, and (v) the amelioration of insulin resistance, with corresponding enhancement of glucose metabolism. In ZFs, excessive visceral adiposity, increased macrophage infiltration and the elevated levels of 8-isoprostane, MIP-1*α*, MCP-1, TNF-*α*, IL-6, IL-1*β*, and ET-1 proteins of heart failure, and extracellular-matrix deposition are among the complex molecular processes that characterize the intricate relationship between inflammation, oxidative stress, cardiac fibrosis, and the progressive development of insulin resistance and cardiomyopathy [5, 47–49, 55–57]. Importantly, the present study unveils that hemin therapy selectively enhances the anti-inflammatory macrophage M2 phenotype in left-ventricular tissue of ZFs while concomitantly abating the proinflammatory M1 phenotype, suggesting that a novel mechanism by which hemin therapy suppresses cardiac inflammation in obesity is by selectively favoring the polarization of macrophage towards the M2 phenotype that ablates inflammation. Correspondingly, hemin therapy abated several chemokines and cytokines that promote macrophage infiltration including MIP-1*α*, MCP-1, TNF-*α*, IL-6, and IL-1*β* [9–11]. Interestingly, the suppression of visceral adiposity and inflammation in hemin-treated ZFs was accompanied by reduced insulin resistance and improved glucose intolerance, and the potentiation of important components of the insulin signal transduction pathway including IRS-1, PI3K, and GLUT4, which in addition to the hemin-dependent enhancement of adiponectin, an anti-inflammatory, insulin sensitizing, and cardioprotective protein [52, 53] may account for improved glucose metabolism in obese conditions.

Hemin therapy also reduced LV hypertrophy, cardiac fibrosis, and cardiomyocyte longitudinal muscle-fiber thickness, a pathophysiological feature of cardiomyocyte hypertrophy [35], with a corresponding suppression of markers of heart failure such as osteopontin and osteoprotegerin [14, 15], as well as the reduction of extracellular matrix protein like TGF-*β*, fibronectin, and collagen which are implicated in cardiac hypertrophy and fibrosis [49, 57]. Since TGF-*β* mobilizes the extracellular matrix by stimulating fibronectin and collagen causing tissue damage and hypertrophy [49, 57], the concomitant reduction of TGF-*β*, fibronectin, and collagen IV in ZFs may account for reduced cardiac lesions. Another mechanism by which the HO system suppresses extracellular matrix and profibrotic agents like TGF-*β* and ET-1 may be due to the HO-dependent potentiation of ANP, a substance known to suppress extracellular matrix [41, 58]. Generally, ANP and ET-1 have opposing effects [59]. For example, ANP reduces fibrosis by inhibiting TGF-*β*1 and fibronectin [41], while ET-1 acts in conjunction with TGF-*β*1 to stimulate the synthesis of fibronectin [58]. Similarly, ANP suppresses inflammation to reduce insulin resistance [59], while ET-1 stimulates inflammatory/oxidative insults causing insulin resistance [60]. On the other hand, ANP stimulates the production of adiponectin [61], a protein with insulin-sensitizing and anti-inflammatory effects [52]. The effects of ANP are largely mediated by cGMP [62], and adiponectin is also known to enhance cGMP [63]. Moreover ANP and the HO system have mutual stimulatory effects. Accordingly, ANP enhances HO [64, 65], and similarly, the HO system has been shown to upregulate ANP and adiponectin [23, 66]. Therefore, the synergistic potentiation of the HO-adiponectin-ANP axis and insulin signaling with corresponding ablation of extracellular matrix/heart failure proteins, the reduction of oxidative stress, and inflammation mediators such as macrophage M1 phenotype, MIP-1*α* and MCP-1, TNF-*α*, IL-6, IL-1*β*, ET-1, and 8-isoprostane are among the multifaceted mechanisms by which hemin therapy improved cardiac function. Thus, novel strategies capable of potentiating the HO-adiponectin-ANP axis would improve cardiomyopathy and insulin signaling in obesity.

Cardiomyocyte hypertrophy and myocardial fibrosis are early microscopic changes in heart failure. Subsequently, macroscopic alterations including increased left-ventricular wall thickness, diastolic/systolic dysfunction, and impaired cardiac hemodynamics become evident. Interestingly, hemin therapy modulated several hemodynamic and echocardiographic parameters to improve cardiac function [67]. These include the reduction of left-ventricular diastolic wall thickness, left-ventricular systolic wall thickness, mean-arterial pressure, arterial-systolic pressure, arterial-diastolic pressure, left-ventricular developed pressure, +dP/dt, and total-peripheral resistance, with corresponding enhancement of stroke volume and cardiac out, and thus improved cardiac function in hemin-treated ZFs. Hemin therapy also enhanced the HO system, cGMP, adiponectin, and ANP, abated 8-isoprostane, MCP-1, MIP-1*α*, TNF-*α*, IL-6, IL-1*β*, and ET-1 and lowered HOMA-IR index in ZL-control rats although the magnitude was smaller as compared to ZFs with depressed HO activity. The reasons for this selective effect of HO are not fully understood. However, it is possible that as ZL controls are healthy animals with normal/functional insulin-signalling, the HO system may be more stable as compared to ZFs which have deregulated HO system with depressed HO-1 and HO activity. Importantly, the selectivity of the HO system in diseased conditions could be explored against the comorbidity of insulin-resistant diabetes and obesity.

Although we recently reported the cardioprotective effects of the HO system in ZDFs [13], a model characterized by obesity, insulin resistance, and overt hyperglycemia, pathophysiological profile of ZDF is not reflective of the metabolically healthy individuals who are characterized by obesity and normoglycemia [7]. In contrast, ZFs closely mimic the pathophysiological profile of metabolically healthy obese individuals with normoglycemia, so our findings may be applicable to this subtype of obese individuals. Moreover, with the rising incidence of cardiometabolic complications in many adults who previously manifested the metabolically healthy obese phenotype [7], novel studies with animal models that closely mimic the pathophysiological profile of metabolically healthy obese subtype are needed. Therefore, studying the effects of the HO system on ZFs may offer new perspective in the pathophysiology of cardiometabolic complications and especially the progressive deterioration of cardiac function which may eventually lead to heart failure given the elevated levels of proteins of heart failure detected in untreated ZFs.

Collectively, our study unveils the beneficial effect of upregulating the HO system in the comorbidity of obesity and insulin resistance and suggests that the suppression of oxidative mediators, macrophage-M1-phenotype infiltration and extracellular matrix/remodeling proteins are among the multifaceted mechanisms by which the HO system maintains and enhances insulin signaling and counteract diabetic cardiomyopathy. These data suggest that although ZFs are normoglycemic, perturbations of insulin signaling, and cardiac function may be forerunners to overt hyperglycemia and heart failure in obesity.

## 5. Conclusion

The novelty of our study includes: (i) the hemin-induced selective enhancement of the anti-inflammatory M2 phenotype in left-ventricular tissue of ZFs and parallel reduction of the proinflammatory macrophage M1 phenotype and MIP-1*α*, a chemokine implicated in macrophage infiltration; (ii) the hemin-dependent suppression of heart failure proteins such as osteopontin and osteoprotegerin; (iii) the suppression of inflammatory cytokines in ZFs; and (iv) the hemin-induced reduction of insulin resistance and improvement of cardiac function in ZFs. Since we recently reported that the HO system suppressed pericardial adiposity in a model characterized by obesity, insulin resistance, and overt hyperglycemia [13], and the present study indicates that hemin therapy abates cardiac inflammation in obesity, an interorgan crosstalk of inflammatory mediators between the myocardium and pericardial adipose tissue can be envisaged in the pathophysiology of diabetic cardiomyopathy. Importantly, the concomitant modulation of macrophage polarization in left ventricles towards the anti-inflammatory M2 phenotype alongside the parallel reduction of proinflammatory cytokines and chemokines implicated in macrophage infiltration and tissue destruction may be indicative of a putative interorgan crosstalk and the movement of inflammatory mediators from the pericardial fat to the myocardium or vice versa, and this may be particularly important in the progressive development of cardiomyopathy, insulin resistance, and related cardiometabolic complications. Although this linkage has to be established, this study would set the stage for further exploration of the putative interorgan communication between pericardial fat and the heart.

## Figures and Tables

**Table 1:** Effect of hemin and stannous mesoporphyrin (SnMP) on physiological and biochemical variables in Zucker fatty (ZF) and Zucker lean (ZL) rats.

Parameters	Animal groups					
	ZL control	ZL + hemin	ZF control	ZF + hemin	ZF + hemin + SnMP	ZF + vehicle
Body weight (g)	472.9 ± 9.7	445.5 ± 11.6^†^	746.8 ± 21.5^§^	685.9 ± 14.7^§^	691.4 ± 15.2^$^	702.8 ± 24.6
Fasting glucose (mmo/L)	7.2 ± 0.6	6.5 ± 0.3*	8.2 ± 0.5^§^	6.9 ± 0.4*	8.5 ± 0.4*	8.1 ± 0.4
Heart weight (g)	1.5 ± 0.03	1.1 ± 0.02^†^	3.4 ± 0.06^§^	2.0 ± 0.04^§^	3.0 ± 0.03^$^	3.1 ± 0.04
Cardiac hypertrophy (g/Kg body weight)	3.1 ± 0.07	2.5 ± 0.06*	4.6 ± 0.16^§^	2.9 ± 0.08*	4.4 ± 0.17*	4.4 ± 0.09

^†^ *P* < 0.05 versus ZL; ^$^ *P* < 0.05 versus ZF; ^§^ *P* < 0.05 versus ZL control; *P < 0.05 versus ZF-control or ZL-control.

**Table 2:** Effect of hemin therapy on hemodynamic and echocardiographic parameters.

Parameters	Experimental groups			*P* value		Effect of hemin on ZF
	ZF	ZL	ZF + hemin	ZF versus ZL	ZF versus ZF + hemin	
Arterial systolic pressure (mmHg)	153 ± 5.2	124 ± 3.4^#^	134 ± 6.3*	0.001	0.018	Reduced by 12.4%
Arterial diastolic pressure (mmHg)	109 ± 3.2	92 ± 2.5^#^	96 ± 4.7*	0.003	0.024	Reduced by 11.9%
Mean arterial pressure (mmHg)	123 ± 3.8	102 ± 2.7^#^	109 ± 5.2*	0.002	0.022	Reduced by 11.4%
Total peripheral resistances (mmHg·min/mL)	1.7 ± 0.1	1.4 ± 0.1	1.4 ± 0.1	0.060	0.080	Reduced by 17.6%
LV developed pressure (mmHg)	161 ± 4.1	136 ± 8.0^#^	140 ± 5.8*	0.012	0.030	Reduced by 13.0%
+dp/dt_(max)_ (mmHg/sec)	3634 ± 127	3050 ± 200^#^	3050 ± 169*	0.027	0.027	Reduced by 16.1%
Heart rate (beats/min)	328 ± 9.2	331 ± 7.2	289 ± 14.2*	0.814	0.020	Reduced by 11.9%
LV diastolic wall thickness (mm)	2.7 ± 0.1	1.7 ± 0.1^#^	1.8 ± 0.1*	0.0001	0.0001	Reduced by 33.3%
LV systolic wall thickness (mm)	3.2 ± 0.1	2.1 ± 0.1^#^	2.7 ± 0.1*	0.0001	0.007	Reduced by 15.6%
LV end diastolic volume (mL)	0.33 ± 0.01	0.40 ± 0.04	0.38 ± 0.02	0.075	0.169	Increased by 15.2%
LV end systolic volume (mL)	0.11 ± 0.01	0.16 ± 0.01^#^	0.14 ± 0.01	0.001	0.089	Increased by 27.3%
Stroke volume (mL)	0.22 ± 0.01	0.24 ± 0.02	0.25 ± 0.02	0.564	0.311	Increased by 13.6%
Cardiac output (mL/min)	72.8 ± 3.6	78.9 ± 9.0	81.8 ± 9.8	0.519	0.412	Increased by 12.4%

Values for each echocardiography and hemodynamic endpoint were averaged for each rat and the mean values used in statistical analyses, with *n*: number of rats. Differences among treatment groups were compared using 1-way ANOVA followed by Fisher's Least Square Difference (LSD) posteriori tests. Differences of *P* < 0.05 were considered statistically significant. Values are means ± SE; *n* = 6 per group. *P < 0.05 versus control ZF rats, ^#^ *P* < 0.05 versus control ZL rats.

LV: left ventricle; +dp/dt_(max)_, maximal rate of increase in left ventricular pressure.
